# Immunization Strategies Producing a Humoral IgG Immune Response against Devil Facial Tumor Disease in the Majority of Tasmanian Devils Destined for Wild Release

**DOI:** 10.3389/fimmu.2018.00259

**Published:** 2018-02-19

**Authors:** Ruth Pye, Amanda Patchett, Elspeth McLennan, Russell Thomson, Scott Carver, Samantha Fox, David Pemberton, Alexandre Kreiss, Adriana Baz Morelli, Anabel Silva, Martin J. Pearse, Lynn M. Corcoran, Katherine Belov, Carolyn J. Hogg, Gregory M Woods, A. Bruce Lyons

**Affiliations:** ^1^Menzies Institute for Medical Research, University of Tasmania, Hobart, TAS, Australia; ^2^Faculty of Science, School of Life and Environmental Sciences, The University of Sydney, Sydney, NSW, Australia; ^3^Centre for Research in Mathematics, School of Computing, Engineering and Mathematics, Western Sydney University, Penrith, NSW, Australia; ^4^School of Biological Sciences, University of Tasmania, Hobart, TAS, Australia; ^5^Save the Tasmanian Devil Program, Tasmanian Department of Primary Industries, Parks, Water and the Environment, Hobart, TAS, Australia; ^6^CSL Ltd., Bio21 Institute, Melbourne, VIC, Australia; ^7^The Walter and Eliza Hall Institute of Medical Research, Melbourne, VIC, Australia; ^8^Department of Medical Biology, The University of Melbourne, Melbourne, VIC, Australia; ^9^School of Medicine, University of Tasmania, Hobart, TAS, Australia

**Keywords:** Tasmanian devil facial tumour disease, vaccination, adjuvant, humoral immunity/antibody response, wild immunology

## Abstract

Devil facial tumor disease (DFTD) is renowned for its successful evasion of the host immune system. Down regulation of the major histocompatabilty complex class I molecule (MHC-I) on the DFTD cells is a primary mechanism of immune escape. Immunization trials on captive Tasmanian devils have previously demonstrated that an immune response against DFTD can be induced, and that immune-mediated tumor regression can occur. However, these trials were limited by their small sample sizes. Here, we describe the results of two DFTD immunization trials on cohorts of devils prior to their wild release as part of the Tasmanian Government’s Wild Devil Recovery project. 95% of the devils developed anti-DFTD antibody responses. Given the relatively large sample sizes of the trials (*N* = 19 and *N* = 33), these responses are likely to reflect those of the general devil population. DFTD cells manipulated to express MHC-I were used as the antigenic basis of the immunizations in both trials. Although the adjuvant composition and number of immunizations differed between trials, similar anti-DFTD antibody levels were obtained. The first trial comprised DFTD cells and the adjuvant combination of ISCOMATRIX™, polyIC, and CpG with up to four immunizations given at monthly intervals. This compared to the second trial whereby two immunizations comprising DFTD cells and the adjuvant combination ISCOMATRIX™, polyICLC (Hiltonol^®^) and imiquimod were given a month apart, providing a shorter and, therefore, more practical protocol. Both trials incorporated a booster immunization given up to 5 months after the primary course. A key finding was that devils in the second trial responded more quickly and maintained their antibody levels for longer compared to devils in the first trial. The different adjuvant combination incorporating the RNAase resistant polyICLC and imiquimod used in the second trial is likely to be responsible. The seroconversion in the majority of devils in these anti-DFTD immunization trials was remarkable, especially as DFTD is hallmarked by its immune evasion mechanisms. Microsatellite analyzes of MHC revealed that some MHC-I microsatellites correlated to stronger immune responses. These trials signify the first step in the long-term objective of releasing devils with immunity to DFTD into the wild.

## Introduction

The Tasmanian devil is the largest living carnivorous marsupial species and is unique to Australia’s island state of Tasmania. The species was listed as Endangered in 2008 due to mortality from devil facial tumor disease (DFTD) (1). DFTD is a fatal transmissible cancer whereby the cancer cells are the infectious agent and pass between individual devils by biting behavior. The cancer’s ability to evade the host’s immune response as it acts as an allograft has been the subject of ongoing research. The devil’s immune system has demonstrated competence from both humoral and cell-mediated perspectives (2–5). The DFTD cancer cells’ ability to avoid an allogeneic immune response is, therefore, not considered due to a defective devil immune system, but rather due to DFTD’s immune escape mechanisms (6, 7). While DFTD immunology and the marsupial devil immune system are in themselves fascinating research topics, the insights gained from such research have a practical application for DFTD vaccine development. A protective vaccine against DFTD would provide an extremely useful tool for managing the endangered species and may help prevent DFTD-driven extinction of the wild devil.

As previously mentioned, successful transmission of DFTD between individuals is considered to be primarily due to the tumor’s immune escape mechanisms. A primary mechanism is the down regulation of the major histocompatibility class I molecule (MHC-I) on the DFTD cell surface (8). Despite this, naturally occurring immune responses against DFTD have been identified in a small number of wild devils (9). Furthermore, immunization trials have demonstrated that humoral and cell-mediated immune recognition of DFTD can be induced (10). Subsequent trials found these immune responses could lead to immune-mediated rejection of the tumors (11). These trials used DFTD cells manipulated to express surface MHC-I as the antigenic basis of the vaccine. This approach was intended to make the tumor cells immunogenic and, therefore, increase the likelihood of raising both antibody and allospecific T-cell responses. However, limitations of these trials included small sample sizes and senescent individuals. The opportunity to address these shortcomings arose with the implementation of the Wild Devil Recovery project by the Tasmanian state government’s Save the Tasmanian Devil Program (STDP; http://www.tassiedevil.com.au/tasdevil.nsf/Wild-Devil-Recovery/8A632773F33E4920CA257EC9001912CE). The ongoing project involves the release of devils from the STDP’s captive insurance and DFTD-free island populations to augment local wild devil populations that have been decimated by DFTD.

The first wild release took place in September 2015 in Narawntapu National Park (NNP) in Tasmania’s north. The devils selected for release were held in free range enclosures (FREs) for several months prior to the release date and 19 were included in this first DFTD immunization trial. The selection of the immunization protocol for this trial was based on results from the previously mentioned trial whereby DFTD cells manipulated *in vitro* to express MHC-I on the cell surface were used as the antigenic basis for the immunizations.

A second release of 33 devils in Stony Head (SH) in the state’s north east took place in August 2016. The SH immunization protocol was shortened in light of the NNP trial results. It also incorporated an improved adjuvant combination that was identified between the NNP and SH releases (12).

Post release monitoring at NNP and SH was carried out by the STDP. Not all devils were trapped following release, but those that had serum samples collected to assess the duration of their anti-DFTD immune responses. Booster immunizations were given to the SH devils that were trapped during the final month of monitoring, which was 5 months after the primary immunization course.

These immunization trials provided the first opportunity to use comparatively large sample sizes. This meant a more robust assessment of anti-DFTD immune responses in Tasmanian devils, as determined by seroconversion, could be made. The responses generated by the different protocols used in the NNP and SH trials with respect to the number of immunizations given and adjuvant combination could also be compared.

Since the initiation of these trials, a second DFTD was discovered (13) and named DFT2 to distinguish it from the first DFTD, which is now referred to as DFT1. The work described here refers to DFT1.

## Materials and Methods

### Tasmanian Devils

There were 19 devils in the NNP trial and 33 devils in the SH trial. All of the NNP devils came from the captive insurance population. Eleven of these NNP devils were originally born in the wild and brought into captivity at the age of 1 year (*N* = 10) or 2 years (*N* = 1). They were quarantined for a period of 30 months to ensure that they were disease free. The other eight NNP devils were born in captivity. Of the SH devils, 16 were born in captivity as part of the captive insurance population. The remaining 17 of the SH devils were from Maria Island, the DFTD-free island population. See Table 1 for age and sex details of the trial devils.

**Table 1:** Summary of devil age, sex, and immunization protocols for Narawntapu National Park (NNP) and Stony Head (SH) trials.

Age	1 year	2 years	3 years	4 years	5 years	Total
**NNP devils**						

No. of males	4	0	0	7	0	11
No. of females	1	1	2	3	1	8

**SH devils**						

No. of males	1	13	8	0	0	22
No. of females	0	1	6	4	0	11

**Complete NNP immunization protocol**						

**Primary course (four immunizations given at monthly intervals)**						
Date of each immunization		Composition of immunizations^a^				
1st: February 2015		2 × 10^7^ MHC-I^+ve^ sonicated cells				
2nd: March 2015		2 × 10^7^ MHC-I^+ve^ sonicated cells				
3rd: April 2015		2 × 10^6^ MHC-I^+ve^ irradiated cells				
4th: May 2015		2 × 10^6^ MHC-I^+ve^ irradiated cells				
**Booster immunization**						
Date of booster		Composition of booster immunization^a^				
September 2015 (pre-release)		2 × 10^6^ MHC-I^+ve^ irradiated cells				

**Complete SH immunization protocol**						

**Primary course (2 immunizations given at monthly intervals)**						
Date of each immunization		Composition of immunizations^b^				
1st: June 2016		2 × 10^7^ MHC-I^+ve^ sonicated cells				
2nd: July 2016		2 × 10^6^ MHC-I^+ve^ irradiated cells				
**Booster immunization**						
Date of booster		Composition of booster immunization^b^				
December 2016 (post release)		2 × 10^6^ MHC-I^+ve^ irradiated cells				

*^a^The combination of adjuvants used in each immunization and booster was as follows: 50 µl ISCOMATRIX™ (provided by CSL Ltd., VIC, Australia), 100 µg polyIC (Sigma-Aldrich, P1530), 50 µg CpG-ODN-1585 (GeneWorks, 1141231), and 50 µg CpG-ODN-2395 (GeneWorks, 1141232)*.

*^b^The combination of adjuvants used in each immunization and booster was as follows: 50 µl ISCOMATRIX™, 100 µg polyICLC (Hiltonol^®^, Oncovir Inc., lot PJ215-1-10-01), and 100 µg Imiquimod (Sigma-Aldrich, 15159)*.

### Devil Enclosures, Trapping, and Blood Sample Collection

The NNP devils were kept in two 11 ha FREs for at least 8 months prior to their release. Males and females were kept separately. The devils were trapped fortnightly during the primary 3-month immunization course. Blood samples were collected each time. For the 4 months prior to the booster immunization, the devils were monitored weekly with camera traps by STDP staff. Blood samples were collected 2 weeks after the booster and a week later the devils were released. The SH devils were kept in two FRE’s, one 11 ha and one 22 ha, for 14 weeks prior to release, and sexes were not separated. They were trapped on three occasions while in the FRE’s for blood collection and immunization.

For each trial, not all devils were trapped each time, and consequently there were some differences in the immunization protocols given, and the blood samples available. Traps were set in each FRE the afternoon before procedures were performed (body weight, physical examination, blood collection, and immunization if required). The traps were baited with possum or lamb flaps and checked the following morning. Each trapped devil was transferred into a hessian sack and the handling and procedures were carried out by two veterinarians/devil keepers. General anesthesia was given in the rare event of not being able to handle the devil in the sack. Devils were released into the FRE immediately following the procedures. Blood sample collection and general anesthesia were performed as described in Ref. (11).

### Vaccine Protocol and Preparation

Devil facial tumor disease immunizations were pre-prepared by treating C5065 DFTD cells with recombinant devil interferon gamma (IFN-γ) [produced by the Walter and Eliza Hall Institute for Medical Research as described in Ref. (11)] diluted 5,000× in culture medium for 24 h. This was to upregulate cell surface expression of the MHC-I molecule (8), and these cells are referred to as MHC-I^+ve^ DFTD cells. The non-manipulated DFTD cells, i.e., cells not expressing surface MHC-I, are referred to as MHC-I^-ve^ DFTD cells. After treatment, MHC-I^+ve^ DFTD cells were inactivated by either four ultrasonic cycles at 50% power on an ultrasonic cell disruptor (sonication) (Misonix Inc., Farmingdale, NY, USA), or by two doses of 40 Gy gamma radiation 24 h apart using a Varian Clinac 23-EX linear accelerator (irradiation) (Varian Medical Systems Inc., Palo Alto, CA, USA).

One day of travel was required prior to the administration of the immunizations. On the morning of the travel day, sonicated preparations in 1 ml phosphate buffered saline (PBS) were taken from −80°C, thawed and adjuvants added. Irradiated cell preparations were thawed, washed twice (with PBS at 500 *g* for 5 min), counted and resuspended in 1 ml, and adjuvants added. The composition of the immunizations, including adjuvants, is detailed in Table 1. The immunizations were kept on ice or at 4°C for 24 h prior to administration. Immunizations were given as a subcutaneous injection between the devils’ scapulae.

### Serum Antibody Detection

**Figure 1:**
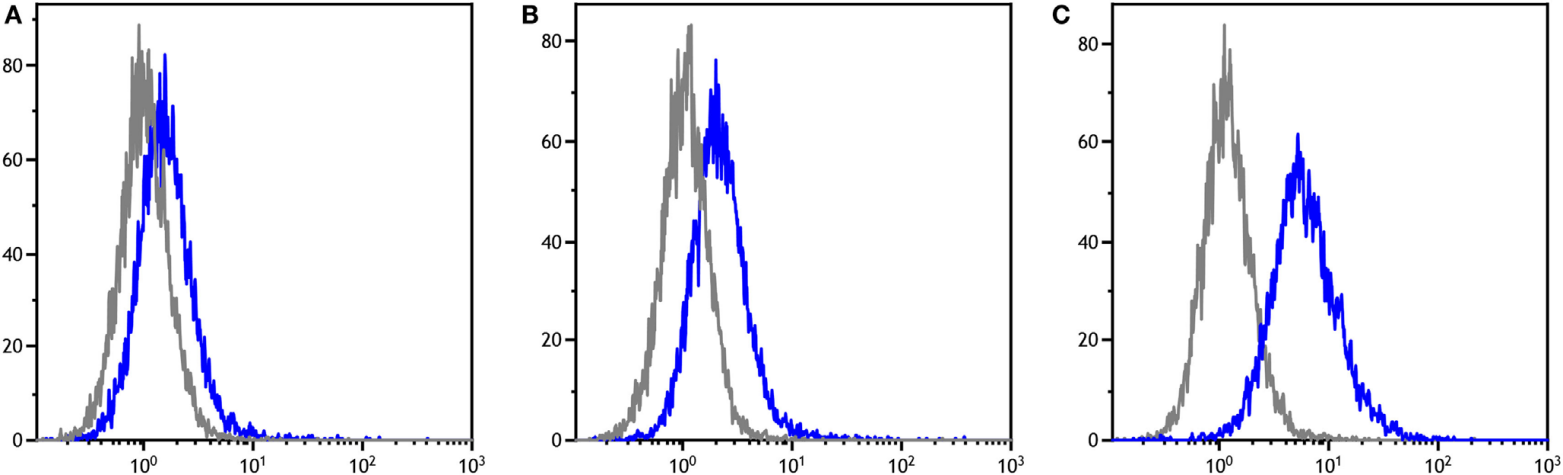
Flow cytometry histograms for three individual devils showing antibodies for MHC-I^-ve^ devil facial tumor disease cells in their pre-immune (gray) and post-immune (blue) serum samples. The fluorescence intensity is in log scale and shown on the *x*-axis, the cell count is on the *y*-axis. **(A–C)** demonstrate the range of responses found across the cohorts.

Indirect immunofluorescence and flow cytometry to measure serum anti-DFTD IgG antibody levels were performed on the serum samples against MHC-I^-ve^ DFTD cells and MHC-I^+ve^ DFTD cells. Preparation of MHC-I^+ve^ cells was described in the Section “Vaccine Protocol and Preparation,” and this and the serum antibody detection method are also described in Ref. (11). In brief, DFTD cells were washed twice with PBS (500 *g*, 5 min). Pre-immune and immune serum samples were diluted 1:50 with washing buffer and mixed with DFTD cells for 1 h. Cells were washed twice with washing buffer, and incubated with 50 µl of 10 µg/ml of a monoclonal mouse anti-devil IgG (provided by the Walter and Eliza Hall Institute for Medical Research) for 30 min, then washed and incubated with 50 µl of 2 µg/ml Alexa Fluor 647 conjugated goat anti-mouse IgG antibody (Life Technologies, A21235) for 30 min. After washing, cells were resuspended in 200 µl of washing buffer containing 200 ng/ml of the cell viability dye 4′,6-Diamidino-2-Phenylindole, Dilactate (Sigma-Aldrich, D9542). Data acquisition was performed on a BD FACSCanto™ II flow cytometer (BD Biosciences, Franklin Lakes, NJ, USA). The median fluorescence intensity ratio (MFIR) was used to classify the antibody responses. The MFIR is defined as the median fluorescence intensity (MFI) of DFTD cells labeled with immune serum divided by the MFI of DFTD cells labeled with pre-immune serum. This ratio accounts for any background serum IgG present prior to the immunizations and standardizes the responses between individual devils. Examples of antibody staining patterns are shown in Figure 1.

### Devil Release and Post-Release Monitoring

Prior to the releases, the incumbent devil populations in NNP and SH were each estimated at 18 individuals, with a DFTD prevalence of 15% (Samantha Fox, personal communication 2016). The NNP trial devils were released on 25 September 2015. The monitoring trips included in this analysis were carried out at 2, 6, and 12 weeks post release. Serum was collected from the immunized devils trapped during the monitoring trips. The SH trial devils were released on 30 August 2016. Monitoring was carried out almost continuously during the 4 months post release, and blood was collected when possible. A booster immunization was given to the SH devils that were trapped in December 2016, 5 months after completion of the primary course.

### Serum Antibody Data Analysis of NNP and SH Trials

The NNP trial took place 1 year before the SH trial. The SH immunization protocol was, therefore, a modified version of the NNP protocol, based on the NNP results and the findings of an adjuvant trial that took place prior to the SH trial. The NNP protocol was longer than SH’s and so had more time points from which serum antibody levels were analyzed. The pre-release responses of the NNP and SH trials are presented here separately, and then compared. The post-release antibody responses of both trials follow.

#### Statistical Analysis of Serum Antibody Data

All MFIR values were log transformed prior to analysis.

**Figure 2:**
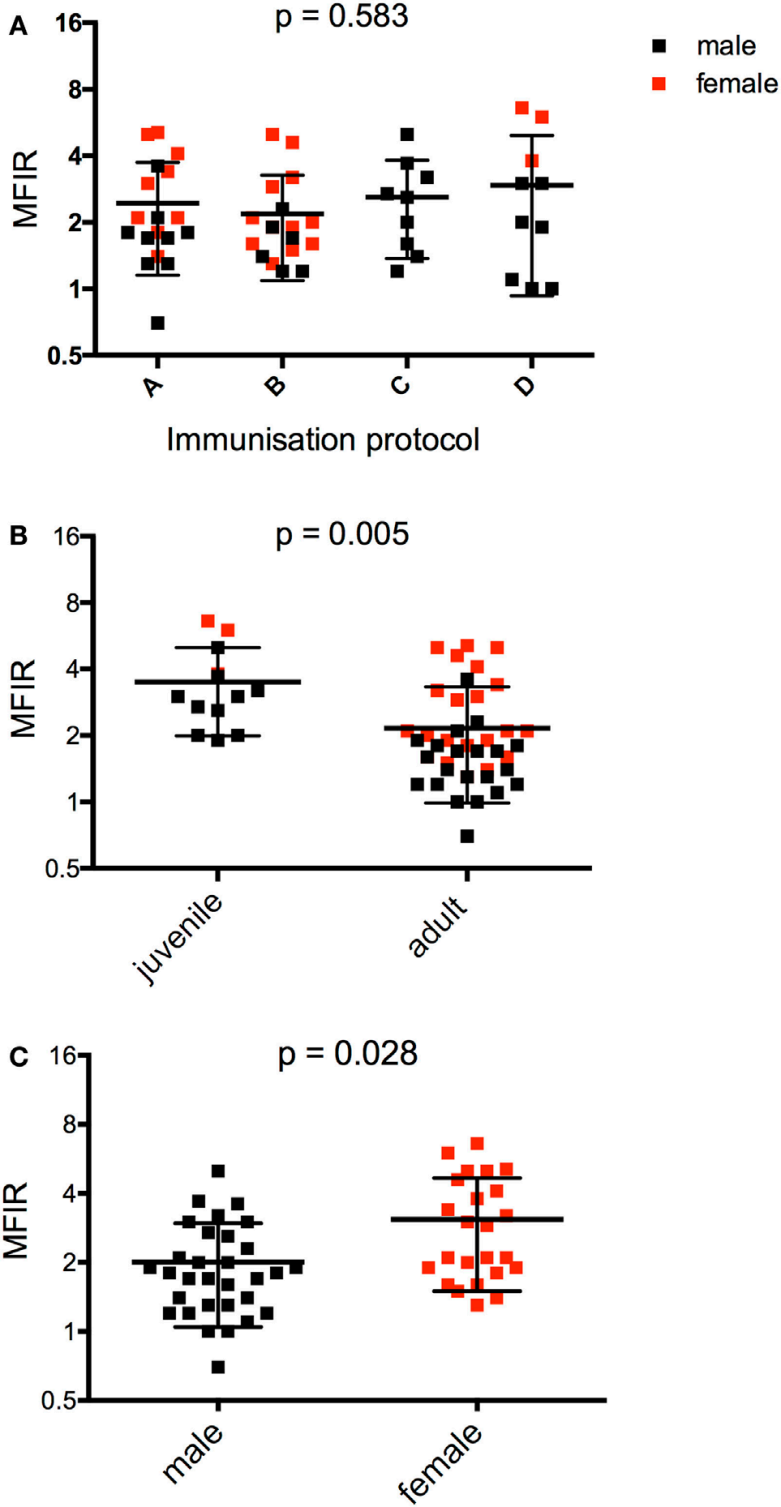
Serum anti-devil facial tumor disease IgG antibody levels (MFIR) for Narawntapu National Park devils showing effect of **(A)** protocol, **(B)** age, and **(C)** sex. The MFIR for each devil at each time point (2 weeks after primary course, on the day of the booster and 2 weeks after the booster) has been plotted on each graph. Protocol A = 4 immunizations at 4-week intervals, B = 4 immunizations at 4- or 6-week intervals, C = 3 immunizations at 4-week intervals, D = 2 immunizations at 2- or 4-week intervals. The *p* values for **(A–C)** were obtained with a four-way ANOVA analysis. See Table 3 for detailed ANOVA results. MFIR, median fluorescence intensity ratio.

**Figure 3:**
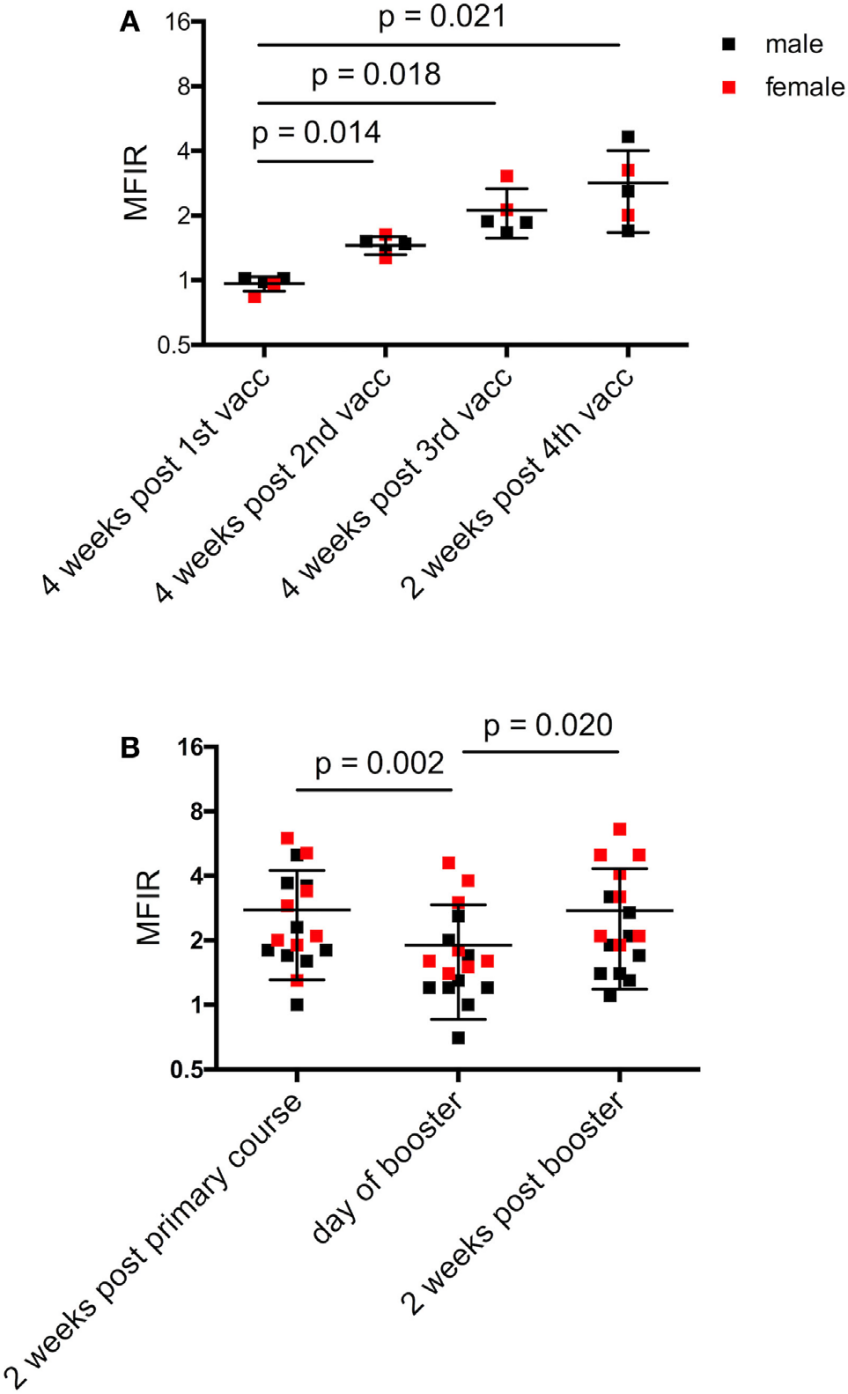
Narawntapu National Park (NNP) devils’ serum anti-devil facial tumor disease IgG antibody levels (MFIR). **(A)** Responses of devils that had all four immunizations in their primary course, i.e., protocol A or B, Table 2. Only those devils for which sera samples were available at all time points are included. **(B)** Responses of all devils for each of three time points: end of primary immunization course, day of booster (4 months later) and 2 weeks post booster. Statistical analysis was performed with repeated-measures one-way ANOVA and only significant *p* values are shown on the graphs. See Table 5 for statistical details. Pre-immune MFIR for each devil is equal to 1 and is, therefore, not shown. MFIR, median fluorescence intensity ratio.

**Figure 4:**
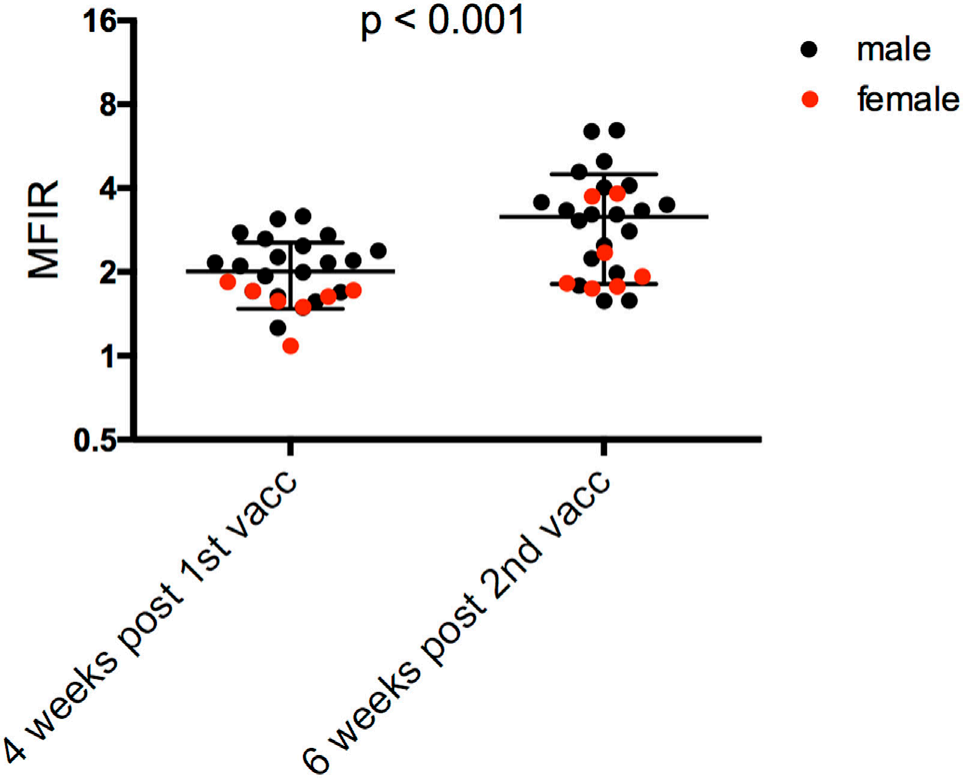
Serum anti-devil facial tumor disease IgG antibody levels (MFIR) of Stony Head devils after their 1st and 2nd immunizations. Pre-immune MFIR for each devil is equal to 1 and is, therefore, not shown. MFIR, median fluorescence intensity ratio.

**Figure 5:**
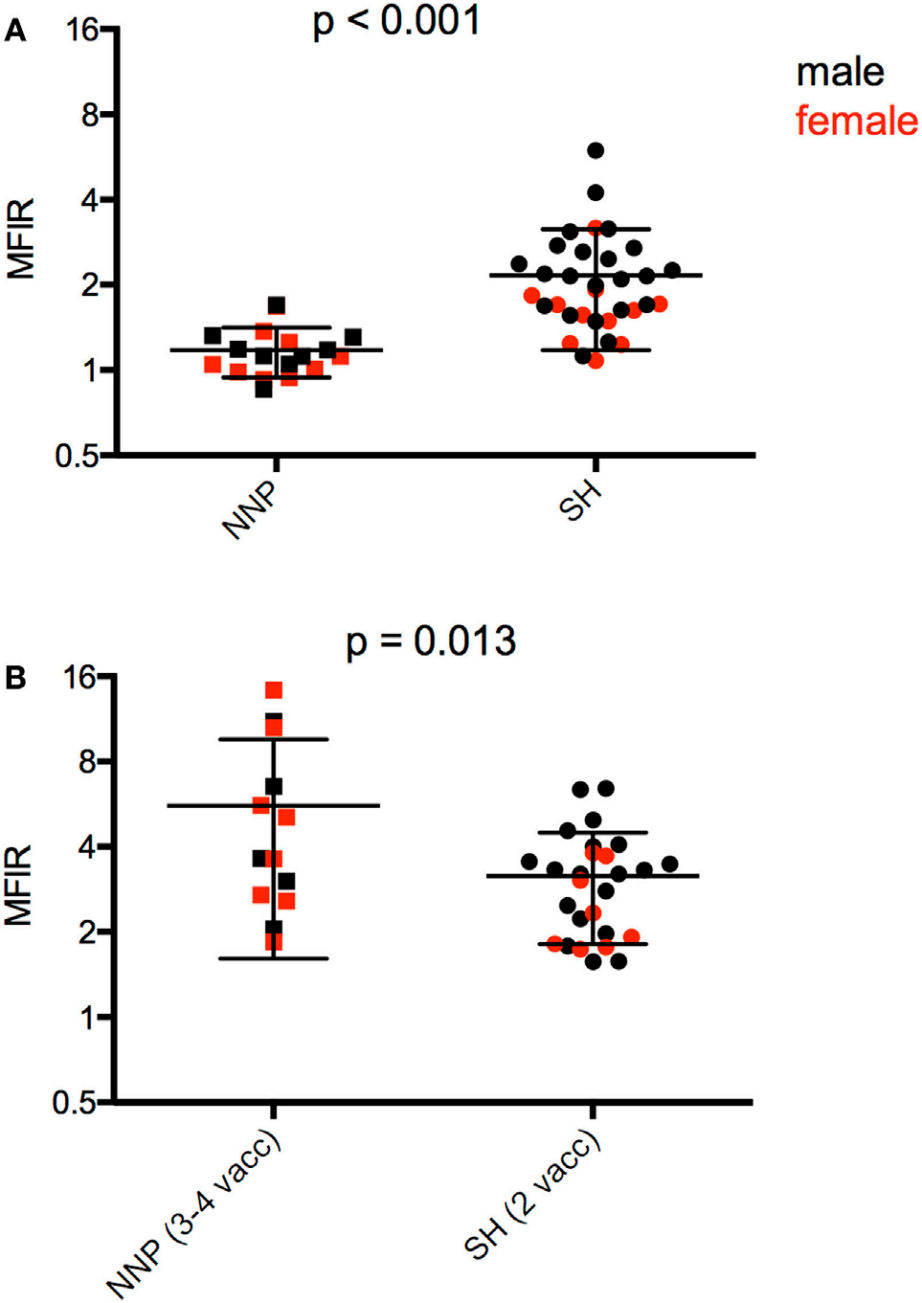
Serum anti-devil facial tumor disease (DFTD) IgG antibody levels [median fluorescence intensity ratio (MFIR)] of **(A)** Stony Head (SH) and Narawntapu National Park (NNP) devils 4 weeks post 1st immunization; **(B)** SH and NNP devils just at the end of their primary immunization courses.

**Figure 6:**
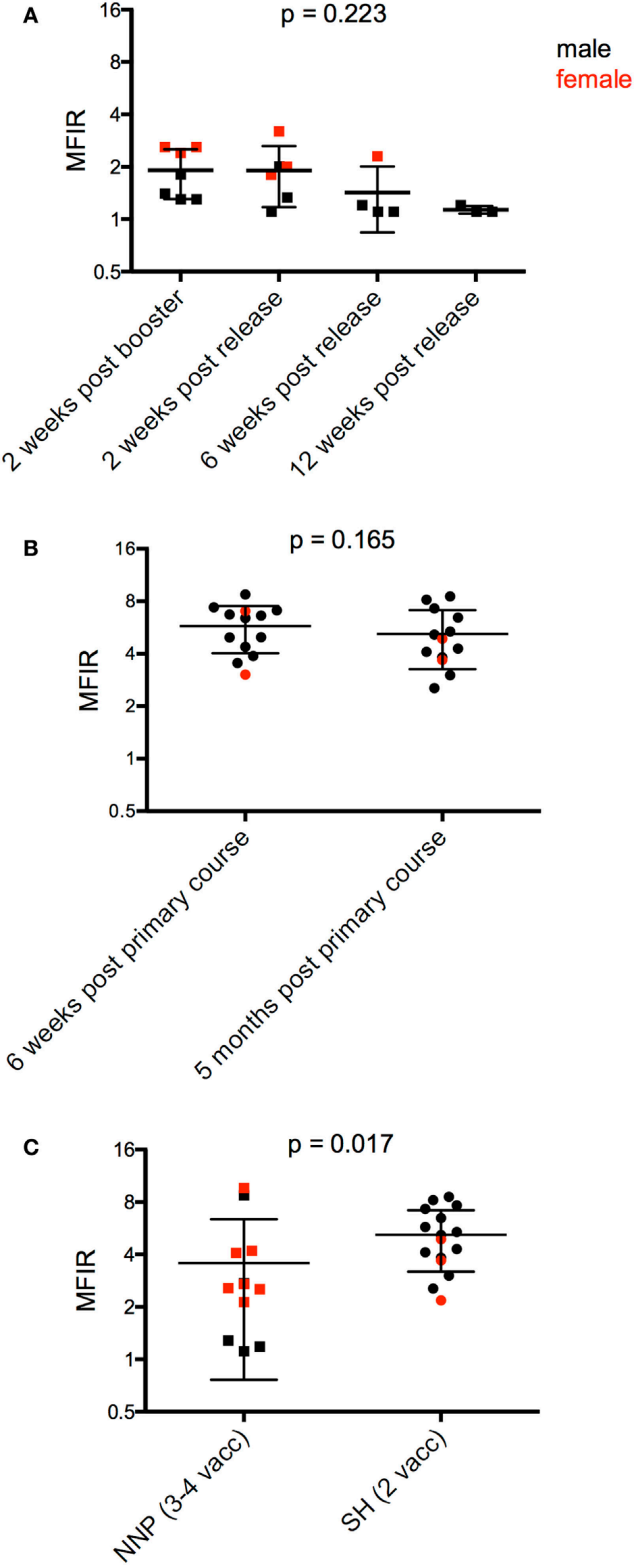
Serum anti-devil facial tumor disease (DFTD) IgG antibody levels [median fluorescence intensity ratio (MFIR)] of **(A)** Narawntapu National Park (NNP) devils post release compared to their post booster response in September 2015; **(B)** Stony Head (SH) devils after their primary course compared to 5 months later; and **(C)** NNP and SH devils, 5 months after their primary immunization course, just prior to the booster administration.

A four-way repeated-measures ANOVA comparing protocol, age and sex over time was performed to compare antibody responses at three time points for the NNP trial (Figure 2). Repeated-measures one-way ANOVAs, paired or unpaired *t*-tests (Figures 3–6) were performed to compare overall anti-DFTD IgG antibody responses at different time periods. Tukey’s *post hoc* analyzes were performed, and for the one-way ANOVAs, multiplicity adjusted *P* values reported.

One-way ANOVAs and t-tests were performed using GraphPad Prism version 6 for Mac OS X, GraphPad Software, La Jolla, CA, USA, www.graphpad.com. The four-way ANOVA was performed using R statistical software (R Core Team 2014).

### MHC Analysis

To determine an association between diversity at MHC-linked microsatellites and individual antibody score, each devil was screened at 12 polymorphic loci (MHC-I 01, 02, 05, 06, 07, 08, 09, 10, 11, 12; MHC-II 02, 03) with devil specific primers (14) (Day, personal communication). Ear biopsies (*N* = 52) were stored in 70% ethanol at −20°C and DNA was extracted using both standard phenol/chloroform protocols and a PureLink Genomic DNA Mini Kit (Thermofisher Scientific, MA, USA). The DNA concentration of each sample was quantified using a Nanodrop 2000 Spectrophotometer (Thermofisher Scientific, MA, USA) and samples normalized to a concentration of 10 ng/µl. Loci were grouped into previously formulated multiplexes (14) determined by the fluorescent tag on either the forward or reverse primer using Multiplex Manager (15).

Polymerase chain reactions (PCR) were performed using the Qiagen Type-it Microsatellite PCR Kit (Qiagen, CA, USA) in a total volume of 10 µl containing 1 × Type-it Multiplex PCR Master Mix (HotStarTaq *Plus* DNA polymerase, Type-it Microsatellite PCR buffer, dNTPs, 2 nM MgCl_2_), 0.2 µM of primer multiplex, and 1 µl of DNA. A negative control using water in place of DNA was included in each 96-well plate run. Products were amplified on a T100 Thermocycler (Biorad, CA, USA) following the manufacturer’s instructions. Amplicon products were sent to AGRF (Westmead, NSW, Australia) for capillary separation using an ABi 3130XL Genetic Analyzer (Applied Biosystems, CA, USA) and scored against the size ladder McLab Orange DMSO 100 (Molecular Cloning Laboratories, SF). Genotypes were assigned to individuals *via* automated allele binning and confirmed visually using GeneMarker 1.95 (Soft Genetics LLC, PA, USA).

#### MHC Statistical Analysis

The association between antibody score and MHC marker was examined using multiple linear regression. Each individual was given a value of 0, 1, or 2 for each allele, depending on the number of copies of the allele that individual carried. In order to adjust for age, sex, and population (NNP or SH), all analyses included these covariates in the models. A model was fitted separately for each marker in MHC-I and MHC-II. The overall significance of a marker was obtained from a likelihood ratio test, where models with/without the marker in question were compared. The effect size of an allele on antibody score are presented as the coefficient from the regression model and its significance was determined using a Wald Test. All analyses were performed using R statistical software (R Core Team 2014).

## Results

### Immunization Protocols

For the NNP trial in particular, the primary immunization protocol each devil received was dependent on trapping success, as well as the time the devils came into the trial. For example, the five juveniles (1-year-old devils) were late entries and, therefore, received only two or three initial immunizations. Table 2 summarizes the different NNP immunization protocols and the number of devils that received each one. All the NNP devils had a booster immunization 4 months after the primary course, just prior to their release.

**Table 2:** Description of Narawntapu National Park (NNP) immunization protocols.

Protocol	Protocol description^a^	Number of devils receiving the protocol
A	4 immunizations at 4-week intervals: 1st and 2nd: sonicated cells 3rd and 4th: irradiated cells	6
B	4 immunizations at 4- or 6-week intervals^b^: 1st and 2nd: sonicated cells 3rd and 4th: irradiated cells	6
C	3 immunizations at 4-week intervals: 1st and 2nd: sonicated cells 3rd: irradiated cells	3 (including 2 juveniles)
D	2 immunizations at 2- or 4-week intervals: 1st: sonicated cells 2nd: irradiated cells	4 (including 3 juveniles)

*^a^See Table 1 for complete description of immunization composition*.

*^b^Two male devils had their 2nd immunizations 6 weeks after the 1st. There were 4-week intervals between their 2nd and 3rd, and their 3rd and 4th immunizations. Four female devils had 4-week intervals between their 1st and 2nd, and 2nd and 3rd immunizations. The 4th immunization was given 6 weeks after the 3rd immunization. Juvenile = 1-year-old devil*.

Of the 33 SH devils, 27 had the primary immunization protocol as outlined in Table 1. There were six devils that had their second immunization on the final pre-release visit since they were not trapped on both the first and second visits. Consequently, there was no blood sample collected after their second immunization and so they were left out of some of the analyses.

### Antibody Responses Prior to Wild Release

The anti-DFTD IgG antibody responses were assessed separately against MHC-I^-ve^ and MHC-I^+ve^ DFTD cells. There were no significant differences between results for the cell types (data not shown), so the results presented below are responses against MHC-I^-ve^ DFTD cells only. This applied to the post release analysis as well. Figure 1 shows representative histograms of the flow cytometry data for serum samples on MHC-I^-ve^ DFTD cells. The negative control for each devil is its pre-immune serum sample and this is compared to the post-immunization serum samples for each individual.

#### Narawntapu Trial

The effects of protocol, age, and sex on the antibody responses measured at three time points were assessed with a four-way ANOVA (Table 3). The primary immunization protocol, whether two, three, or four immunizations, did not make a significant difference to the antibody responses (Figure 2A). However, both age and sex were found to have significant effects on these (Figures 2B,C; Table 3). Juveniles had higher responses on average than adults, and females had higher responses than males.

**Table 3:** Results of statistical tests comparing serum antibody responses for (A) Figure 2.

Four-way ANOVA (Figure 1. Narawntapu National Park: effect of protocol, age, and sex on serum antibody)			

	df	*F*	*P*
Sex	1	6.609	0.028
Age	1	12.417	0.005
Protocol	3	0.682	0.583
Sex:age	1	0.348	0.569
Sex:protocol	1	0.234	0.639
Sex:time	1	3.811	0.061
Age:time	1	0	0.984
Protocol:time	3	0.820	0.494
Sex:age:time	1	1.364	0.253
Sex:protocol:time	1	0.163	0.690
Age:protocol:time	1	1.279	0.268
Error	27		

Figure 3A shows the responses for devils that received four immunizations in the primary course, either protocol A or B, for which serum samples were available at each time point. Antibody levels were significantly higher after the second, third, and fourth immunizations compared to the first (Table 4).

**Table 4:** Results of statistical tests comparing serum antibody responses for (B) Figures 3–6.

Repeated-measures ANOVA					

Figure	df	*F*	*P*	Adjusted*P*values for multiple comparisons (not shown on graphs)	
				Columns^a^	*P*
3 (A) NNP devils that received protocol A or B	3	18.10	0.001	B–C	0.067
				B–D	0.072
				C–D	0.543

3 (B) NNP, both sexes	2	7.061	0.005	A–C	0.986

3 (B) NNP, males	2	19.190	<0.001	A–B	0.004
				A–C	0.035
				B–C	0.003

3 (B) NNP, females	2	9.552	0.008	A–B	0.257
				A–C	0.071
				B–C	0.003

6 (A) NNP, post release compared to post booster^b^	2	2.200	0.223	A–B	0.976
				A–C	0.200
				B–C	0.199

**Figure**	**df**	*****t*****	*****P*****

**Paired *t*-test**					
4. SH after 1st and 2nd immunizations, both sexes	26	5.438	<0.001		
4. SH males	19	4.798	<0.001		
4. SH females	6	2.405	0.053		
6 (B) SH, post primary course compared to time of booster	12	1.480	0.165		

**Unpaired ***t***-test**					
5 (A) SH and NNP, 4 weeks post 1st immunization	49	5.713	<0.001		
5 (B) SH and NNP after primary immunization course	38	2.600	0.013		
6 (C) NNP and SH just prior to booster administration	26	2.540	0.017		

*^a^A, B, C, and D refer to columns in the graphs, i.e., A, 1st column, B, 2nd column; C, 3rd column; D, 4th column*.

*^b^Only 3 time points compared statistically due to missingness*.

*NNP, Narawntapu National Park trial; SH, Stony Head trial*.

Antibody levels of all devils were then compared at three time points: 2 weeks after the primary course; on the day of the booster; and 2 weeks after the booster. At the end of the primary immunization course, only two NNP devils failed to respond (MFIR < 1.5) (Figure 3B). There was a significant difference in MFIR over time (Table 4). The levels on average were lowest on the day of the booster which was 4 months after the primary course. The booster resulted in an increase in antibody levels similar to the level at the end of the primary course (Figure 3B). A sex difference was apparent whereby post booster antibody levels in the adult males did not reach the levels found after the primary immunization course. By contrast, there was a trend for the booster to result in female devils having antibody levels that were equal to or higher than levels achieved after the primary course (Figure 3B; Table 5).

**Table 5:** Devil MHC microsatellite markers that have alleles significantly associated with a high antibody response.

MHC marker	*P*-value of marker (adjusted for age, sex, and population)	Allele	Effect size	*P*-value of allele (adjusted for age, sex, and population)	Mean antibody score (median fluorescence intensity ratio), and number of devils with 1 or 2 copies of the allele	Vaccine cell line alleles
MHC-I 02	0.041^a^	201	0.56	0.048^a^	3.5, *N* = 20	/
		203	NA	NA	2.9, *N* = 40	203, 203

MHC-I 08	0.029^a^	216	0.95	0.195	2.5, *N* = 23	/
		218	1.91	0.054	3.3, *N* = 3	/
		220	1.4	0.069	3.2, *N* = 20	220
		222	1.73	0.030^a^	3.5, *N* = 17	/
		224	NA	NA	1.8, *N* = 3	224

MHC-I 10	0.003^a^	243	3.37	0.039^a^	6.4, *N* = 1	/
		245	0.71	0.535	3.3, *N* = 33	/
		247	0.09	0.938	2.5, *N* = 27	/
		249	NA	NA	1.8, *N* = 1	249
		251	NA	NA	*N* = 0	251

*Models were adjusted for age, sex, and population. Age was found to have a significant association with antibody response. The alleles of the MHC markers significantly associated with high serum antibody are also shown, along with their P values. The effect size indicates the average increase in serum antibody observed for each copy of the allele. P values for alleles were obtained from a likelihood ratio test/Wald test, respectively. The last column shows the alleles of the C5065 cell line used in the vaccine*.

*^a^Indicates significance*.

*NA = not available; / = none shared with respect to MHC allele*.

#### SH Trial

Antibody levels were significantly higher for the SH devils 6 weeks after the second immunization compared to 4 weeks after the first immunization (Figure 4; Table 4). Sex did not have a significant effect on the SH devils’ responses at the end of their primary immunization course. There was only one juvenile devil in the SH trial so a comparison between juvenile and adult responses was not possible.

#### Comparison of SH and NNP Trials

The only directly comparable time point for the NNP and SH trials was 4 weeks after the first immunization. The SH devils had significantly higher serum antibody levels than NNP devils (Figure 5A; Table 4). At the end of the primary course, the NNP devils that had three or four immunizations had higher serum antibody levels than the SH devils (Figure 5B; Table 4).

A the end of their primary course, 17 of the 19 NNP devils, and all 33 of the SH devils had seroconverted providing an overall percentage of 96% for seroconversion in response to the DFTD immunizations.

### Antibody Responses after Wild Release

Following the release into NNP, monitoring trips included in this analysis were carried out at 2, 6, and 12 weeks. There were six devils trapped on the first post monitoring trip, and only three devils at 12 weeks. There was a trend for antibody levels to decrease over this time to almost baseline levels at 12 weeks (Figure 6A; Table 4).

Monitoring at SH was carried out almost continuously for 4 months post release, i.e., up until 5 months after the primary course. There were 17 devils that were trapped, had blood collected, and given a booster immunization in the final month of monitoring. Of these devils, 13 had blood samples collected 6 weeks after their second immunization. Antibody levels were maintained by these 13 devils over the 5-month period between their second immunization and booster (Figure 6B; Table 4). This contrasts with the NNP devils whose antibody levels dropped over a similar time frame (Figure 2B, 2 weeks post primary course compared to at time of booster). Five months after the primary course, the SH devils, which all received a total of two immunizations, had a higher overall antibody level than the NNP devils which had received three or four immunizations (Figure 6C; Table 4).

### MHC Microsatellites Markers

Microsatellites across the MHC-I and MHC-II were compared to the antibody responses (MFIR). Highly significant (*p* < 0.005) associations occurred between antibody responses and MHC-I_10, and a significant association (*p* < 0.05) was identified for MHC-I_02 and MHC-I_08 (Table 5). No significant associations were found with MHC-I loci 01, 05, 06, 07, and 11. There was limited diversity across MHC-II loci (02 and 03) and no associations were identified. The significance of the effect of each MHC-I and MHC-II microsatellite markers on serum antibody are shown in Table S1 in Supplementary Material.

The statistical analysis of the MHC data and its association with antibody response was adjusted for age, sex, and population (NNP or SH). A significant effect of age on antibody score was demonstrated, with an average decrease in antibody score of 0.76 for every year of a devil’s life (*p* < 0.001). There were non-significant effects observed for population (NNP devils having an antibody score of 0.12 greater than SH devils, *p* = 0.77) and sex (females having an antibody score of 0.1 greater than males, *p* = 0.81).

The DFTD tumor cell line used in the immunizations was assessed for the presence of microsatellites. None of the three alleles associated with a higher antibody response were present in the C5065 cell line that was used for the immunizations (Table 5).

## Discussion

These trials brought about the first cohorts of devils immunized against DFTD and released to the wild in the STDP’s Wild Devil Recovery project. The collaboration combined two innovative management strategies to prevent the extinction of devils in the wild: DFTD immunization and population augmentation. The immunization trials were performed on a much larger number of devils than had previously been possible, allowing for a robust assessment of the induced immune responses. The two trials differed with respect to the number and timing of the immunizations given in the primary course, and the adjuvant combination.

There are other examples of vaccinating captive bred and/or wild populations of endangered species against fatal diseases, e.g., black-footed ferrets against plague and canine distemper virus (16, 17), kakapo against erysipelas (18), and Ethiopian wolves against rabies (19, 20). An experimental chlamydia vaccine trial has also been carried out on a wild population of koalas in south east Queensland (21). The DFTD immunization trials shared aspects of these examples, notably a focus on an endangered species facing a primary threat of disease which might be addressed by vaccination. However, most of the previous examples are against microorganisms for which vaccines have been established.

A vaccine protecting against DFTD would be a valuable conservation tool to secure the future of the wild devil population. There are suggestions that responses to DFTD are occurring in the wild. Natural immune responses against DFTD have been found in a small percentage of wild devils (9). There is also evidence that genetic selection associated with DFTD has occurred in certain populations (22). However, there has not been a measurable reduction of the DFTD effect on these populations. DFTD has resulted in dramatic devil population decline to the point where the species is functionally extinct in certain locations (23). Even with the assumption that adequate anti-DFTD responses are evolving, the ecological impacts of a decimated devil population are profound and relying on natural selection of resistant animals for population recovery at this stage seems risky. A protective DFTD vaccine would aid in timelier restoration of a functional devil population while helping to ensure maintenance of the genetic diversity of the species.

With respect to DFTD vaccine development, the biggest advantage of the trials described in this study was the sample size. Previous immunization studies have been carried out on a maximum of four devils at a time. The comparatively large sample sizes here allowed for greater confidence in the assessment of anti-DFTD immune responses. It also allowed for the effects of age, sex, differing immunization protocols, and MHC variation to be evaluated. Prior immunization trials on captive devils have demonstrated that an immune response against DFTD is achievable. However, it was unknown if results could be generalized to the wider devil population due to the small sample sizes. The high number of responders in these trials suggests most devils are capable of producing an immune response against DFTD which is encouraging for vaccine development.

MHC-linked microsatellite analysis of the immunized devils showed that particular MHC alleles were associated with a higher antibody response to the immunizations. These alleles were not present in the tumor cell line that was used as the basis of the immunization. This suggests that the MHC type of the individual devil may influence its response to DFTD immunizations. It also implies that an immune response against DFTD is more likely if the MHC of the devil and the tumor are mismatched. However, there is no evidence in the literature that MHC type affects devil responses to naturally occurring DFTD infection (24, 25). Sequencing analysis of the MHC genes themselves and their expression may provide further insight into the relationship between MHC and a devil’s ability to respond to immunization. IgG responses in both NNP and SH trials were specific for both non-manipulated and MHC-I upregulated DFTD cells, suggesting that immune responses against DFTD surface antigens other than MHC were generated. These responses will be critical for recognition of MHC-I^-ve^ DFTD cells upon exposure to the disease in the wild.

The statistical analysis of the MHC data and its association with antibody response found a significant effect of age. This result is in accordance with previous research showing the decline of the devil’s immune capacity with age (26). The NNP trial included five juvenile devils which allowed for the effect of age (juvenile compared to adult) on the immune response to be assessed. The juveniles showed, on average, higher antibody responses than the adult devils. This was despite the juveniles receiving fewer immunizations in the primary course than the majority of adults. Only one juvenile was in the SH trial, which precluded a comparison between juvenile and adult responses. The NNP trial showed female devils to have significantly higher antibody responses than males. There was no overall significant sex difference found in the SH trial. SH males did, however, show a significant increase in antibody levels after their second immunization, whereas there was only a trend for antibody levels to increase in the females. There is evidence that sex affects immune responses *via* a combination of genetic, hormonal, and environmental factors with human studies generally showing females to have heightened immunity to pathogens, and a tendency toward higher responses to bacterial and viral vaccines than males (27).

Compared to conventional immunization protocols against microbial pathogens, the NNP immunization protocol was long, taking 7 months including the booster prior to the release date. Not all devils received the entire primary immunization course, and results suggested that a reduced number of primary immunizations did not affect the post booster antibody response. This evidence that similar antibody responses could be achieved with fewer immunizations was used to refine the protocol for the SH trial. Similarly, results from an adjuvant study on captive devils that took place prior to the SH trial influenced the choice of adjuvant used in the SH protocol (12). A shorter and, therefore, more practical protocol with a promising adjuvant combination was implemented for SH. Both the NNP and SH trials resulted in similar levels of serum antibody. The improvements identified in the SH trial were that antibody levels were achieved more rapidly and were of longer duration. Following their first immunization, the SH devils had higher overall antibody levels than the NNP devils. These levels in the SH devils were maintained over the 5 months prior to the booster compared to the NNP devils which had a significant decline in antibody levels pre-booster. In addition, the NNP devils trapped 3 months after release had antibody levels approaching baseline. This suggests that superior immune memory was stimulated in the SH devils. It is likely that the adjuvant combination used in the SH trial was responsible for the improved speed and duration of response.

The adjuvant component in both trials included ISCOMATRIX ™, a proprietary saponin-based adjuvant which has previously been shown to generate antigen-specific CD8+ T cells and result in regression of established solid tumors when combined with toll-like receptor (TLR) agonists (28). The adjuvant component of the NNP and SH trials differed in their use of TLR agonists. The NNP trial included the TLR9 agonist, CpG- oligodeoxynucleotide (CpG), and the TLR3 agonist, polyinosinic:polycytidylic acid (polyIC). This combination was in keeping with the DFTD immunization trials that documented tumor regression (11).

As mentioned above, a study was undertaken prior to the SH trial to assess adjuvant effects on the immune responses of devils to the model antigen, keyhole limpet hemocyanin (KLH) (12). The study used the TLR-7 agonist imiquimod, and polyICLC (Hiltonol^®^) which is more stable than polyIC due to its ribonuclease resistance. Imiquimod alone resulted in a minimal immune response to KLH. PolyICLC, however, gave a robust antibody response when used alone and also in combination with imiquimod. In light of these results, the SH trial protocol omitted CpG and replaced it with imiquimod, and the polyIC was replaced with polyICLC.

As discussed in Patchett et al. (12), polyICLC and imiquimod activate potent antigen-specific immunity through the stimulation of multiple immune pathways in human and animal studies. PolyIC results in production of large amounts of type-1 IFN. In mice immunized with polyICLC, substantial increases in CD8^+^ T-cell cytotoxicity occurred (29, 30). Imiquimod is also a potent stimulator of type-1 IFN *via* TLR-7-dependent activation of plasmacytoid dendritic cells (pDCs) (31). Previous studies of adjuvant efficacy have demonstrated improved generation of T-cell memory in response to polyICLC or imiquimod compared to other adjuvants (32, 33). Similar responses in Tasmanian devils to DFTD immunizations containing polyICLC may offer a greater likelihood of protection against DFTD.

There were two notable limitations to these trials. The first was the inability to measure the cell-mediated immune response to the immunizations due to the absence of a reliable cytotoxicity assay. The challenges of designing and implementing accurate T-cell assays in human clinical trials are substantial and well reviewed (34). Furthermore, *in vitro* evidence for specific T-cell responses does not always predict vaccine-mediated protection and Saade et al. highlight the need for better correlates of vaccine-induced T-cell immunity.

Here, serum IgG antibodies specific for DFTD were used to measure the responses induced by the immunizations (11). Since IgG production is T cell dependent, this provides indirect evidence for T-cell involvement. Previous research has suggested that a humoral immune response can inhibit development of a protective cellular immune response (35), the latter being requisite for a cancer vaccine. However, the correlation between anti-DFTD serum antibodies and DFTD regression has been documented in both wild and captive devils (9, 11) indicating the relevance of serum antibodies in an anti-DFTD response. As was postulated in these references, antibody-dependent cell-mediated cytotoxicity is a mechanism by which antibodies could facilitate tumor cell killing.

Another limitation associated with the trials was the low post-release trapping success of immunized devils. This applied particularly to the NNP trial. The low NNP recapture rate was partly due to the devils’ likely dispersal beyond the trap lines, but also due to deaths from road traffic accidents (36). Trapping at SH was more successful due to the SH devils being fitted with GPS collars just prior to their release. The devils were, therefore, trackable until the collars were removed within 4 months of release, and it was possible to collect serum from 17 SH devils 4 months after the release date. Future trapping success will dictate whether the duration of antibody responses and the effect of the immunization protocol on DFTD susceptibility can be confidently ascertained. At the time of publication, three of the devils released at SH have been confirmed positive for DFTD due to natural exposure. Longitudinal sample collection and analysis are required before drawing conclusions on whether the immunization responses influence tumor growth rate in the devils that acquire disease.

Despite the limitations, these trials signify a notable advance in DFTD vaccine research. Although it remains unclear what protection the immunization protocols provided against a natural DFTD challenge, the serum antibodies detected in the majority of devils in response to the immunizations suggest that development of an effective DFTD vaccine is a realistic expectation. The improved antibody response obtained with the shorter protocol used in the SH trial was most likely a function of the adjuvant used and was a particularly encouraging finding for the eventual feasibility of an immune solution to DFTD.

## Ethics Statement

All procedures were approved by the University of Tasmania Animal Ethics Committee (permit number A0014599). Permission to obtain samples was approved by the Tasmanian Department of Primary Industries, Parks, Water and Environment (DPIPWE) for the period 20 September 2016 to 19 September 2021 inclusive.

## Author Contributions

The field work and experiments were conceived and designed by RP, AM, AS, MP, AK, SF, CH, GW, AL, DP, and LC. The laboratory experiments were performed by RP, AP, and EM. The data was analyzed by EM, RT, SC, AP, RP, KB, CH, GW, and AL. The manuscript was written by RP, GW, and AL. All authors approved the manuscript.

## Conflict of Interest Statement

The authors declare that the research was conducted in the absence of any commercial or financial relationships that could be construed as a potential conflict of interest.
