# Supplementary material for: Immunization Strategies Producing a Humoral IgG Immune Response against Devil Facial Tumor Disease in the Majority of Tasmanian Devils Destined for Wild Release
Source: Front Immunol. 2018 Feb 19;9:259. doi: 10.3389/fimmu.2018.00259 (PMC5826075; doi:10.3389/fimmu.2018.00259)
Supplement: Supplementary file 1 [file table_1.docx]

**Supplementary material for:**

**Immunisation strategies producing a humoral IgG immune response against DFTD in the majority of Tasmanian devils destined for wild release**

**Supplementary Table S1.** The significance of the effect of MHC-I and MHC-II microsatellite markers on serum antibody (markers with a significant P value are associated with high antibody responses).

| **MHC markers** | **Chi sq** | **Df** | **P value** |
| --- | --- | --- | --- |
| MHC-I 01 | 6.627 | 3 | 0.194 |
| MHC-I 02 | 5.656 | 1 | 0.041* |
| MHC-I 05 | 4.012 | 1 | 0.091 |
| MHC-I 06 | 6.684 | 2 | 0.087 |
| MHC-I 07 | 9.357 | 8 | 0.639 |
| MHC-I 08 | 13.510 | 4 | 0.029* |
| MHC-I 09 | 3.106 | 2 | 0.346 |
| MHC-I 10 | 16.178 | 3 | 0.003** |
| MHC-I 11 | 5.304 | 5 | 0.627 |
| MHC-I 12 | 3.106 | 2 | 0.346 |
| MHC-II 02 | 2.548 | 3 | 0.641 |
| MHC-II 03 | 1.913 | 4 | 0.876 |
